# Enhanced immunomodulatory effects of canine adipose tissue-derived mesenchymal stem cells in 3D culture

**DOI:** 10.3389/fvets.2025.1500267

**Published:** 2025-03-26

**Authors:** Eunbi Lee, Ga-Hyun Lim, Ju-Hyun An, Min-Ok Ryu, Kyoung-Won Seo, Hwa-Young Youn

**Affiliations:** ^1^Laboratory of Veterinary Internal Medicine, Department of Veterinary Clinical Science, College of Veterinary Medicine, Seoul National University, Seoul, Republic of Korea; ^2^VIP Animal Medical Center, Seoul, Republic of Korea; ^3^Department of Veterinary Emergency and Critical Care Medicine and Institute of Veterinary Science, College of Veterinary Medicine, Kangwon National University, Chuncheon-si, Republic of Korea

**Keywords:** dog, mesenchymal stem cell, 3D culture, spheroid, immunomodulation

## Abstract

**Introduction:**

Mesenchymal stem cells (MSCs) have been introduced as a treatment for dogs owing to their immunomodulatory effects. In humans, 3D-cultured MSCs have recently been applied in treating various conditions, including myocardial infarction, liver disease, and kidney disease. This study aimed to evaluate whether the immunomodulatory effects of canine adipose tissue-derived MSCs (cAT-MSCs) are enhanced when cultured in a 3D environment compared to conventional 2D culture.

**Methods:**

cAT-MSC spheroids were generated using ultra-low-adhesion plates. The structural and hypoxic characteristics of these spheroids were assessed via confocal imaging. The expression levels of the stemness markers SOX2 and OCT4 were examined through western blotting. Additionally, the expression of inflammatory factors within the cAT-MSC spheroids was analyzed using RT-PCR and ELISA. The immunomodulatory effects were further evaluated in canine macrophages (DH82) treated with conditioned media (CM) from cAT-MSC spheroids, using RT-PCR and flow cytometry.

**Results:**

3D culture induced hypoxic conditions within the cAT-MSC spheroids and significantly increased the expression of SOX2 and OCT4 (*p* < 0.05). Moreover, the expression of inflammation-associated factors, including TGF-β_1_, TSG-6, COX-2, PGE2, and IL-10, was upregulated in the 3D culture (*p* < 0.05). Treatment of DH82 cells with CM from the cAT-MSC spheroids led to a significant reduction in the expression of pro-inflammatory factors such as TNF-*α*, IL-1β, and IL-6 (*p* < 0.01). Additionally, M1 polarization was diminished in DH82 cells exposed to the CM from the cAT-MSC spheroids (*p* < 0.0001). And M2 polarization was increased in DH82 cells exposed to the CM from the cAT-MSC spheroids (*p* < 0.0001).

**Conclusion:**

This study confirms that the immunomodulatory effects of MSCs are enhanced in 3D culture. Therefore, 3D cultured MSCs may offer a more effective therapeutic approach than conventional 2D-cultured MSCs for treating canine inflammatory diseases.

## 1 Introduction

Mesenchymal stem cells (MSCs), multipotent stromal cells first isolated from bone marrow (1), have the capacity to differentiate into various cell lineages, including osteoblasts, chondrocytes, and adipocytes, depending on the isolation technique, culturing protocols, and media used (2, 3). These cells are present in a range of tissues, such as bone marrow, adipose tissue, umbilical cord blood, dental pulp, and synovium (4, 5). MSCs perform their therapeutic functions through various mechanisms, including their differentiative potential, immunomodulatory properties, production of therapeutic exosomes, production of growth factors and cytokines, and anti-apoptotic effects (6–8). By modulating immune responses and reducing inflammation, MSC therapies are pivotal in resolving inflammatory conditions (7, 9) and maintaining a balance between pro-inflammatory and anti-inflammatory states (10). Similarly, canine adipose tissue-derived stem cells (cAT-MSCs) are expected to offer therapeutic benefits and have been evaluated in clinical trials for treating conditions such as inflammatory bowel disease and canine atopic dermatitis (11, 12).

Traditional two-dimensional (2D) cell culture methods have long been the standard approach. However, 2D cultures lack the structural complexity and the crucial cell–cell and cell-extracellular matrix (ECM) interactions necessary for normal cell function and differentiation, limiting their ability to accurately reflect the *in vivo* microenvironment (13). These limitations have driven the development of more physiologically relevant models that better mimic *in vivo* conditions. Three-dimensional (3D) culture methods offer a more accurate representation of the stem cell niche by providing spatial organization and enhanced cell–cell interactions (14, 15), which can lead to improved cellular signaling and function. Notably, 3D-cultured MSC spheroids have been shown to possess additional benefits, including enhanced immunomodulatory and tissue regenerative effects (16–18).

In this study, we cultured cAT-MSCs in a 3D environment to assess whether their immunomodulatory effects are enhanced compared to those in conventional 2D cultures. Our objectives were to form cAT-MSC spheroids, evaluate the maintenance of stemness in these spheroids, and determine whether the expression of immunomodulatory factors is increased in cAT-MSC spheroids.

## 2 Materials and methods

### 2.1 Cell culture

cAT-MSCs were cultured in Dulbecco’s Modified Eagle’s medium (DMEM; Welgene, Gyeongsan-si, Korea) supplemented with 10% fetal bovine serum (FBS; Gibco, Billings, MT, United States) and 1% penicillin–streptomycin (PS; PAN-Biotech, Aidenbach, Germany). The canine macrophage cell line DH82 (ATCC number: CRL-10389; Manassas, VA, United States) was cultured in DMEM supplemented with 10% FBS and 1% PS. All cells were incubated at 37°C in a 5% CO_2_ environment. The culture medium was replaced every 2–3 days, and cells were subcultured upon reaching 70–80% confluency.

### 2.2 Isolation and characterization of cAT-MSCs

Canine adipose tissue was collected from three healthy adult female dogs that had undergone ovariohysterectomy at the Seoul National University Veterinary Medical Teaching Hospital (SNU VMTH). This procedure was conducted following a protocol approved by the Institutional Animal Care and Use Committee of SNU (protocol no. SNU-220818-1). MSC isolation and culture were performed as previously described (19). Adipose tissue samples were washed with Dulbecco’s phosphate-buffered saline (DPBS; Welgene) supplemented with 1% PS. The tissue was then minced using sterilized scissors, treated with 0.1% collagenase type IA (Gibco), and incubated at 37°C for 1 h. After incubation, the samples were neutralized with DMEM supplemented with 10% FBS and centrifuged at 1,200 × g for 5 min. The resulting pellet was passed through a 70-μm cell strainer (Thermo Fisher Scientific, Waltham, MA, United States) and centrifuged again at 1,200 × g for 10 min. To remove any remaining blood cells, the pellet was resuspended in RBC lysis buffer (Sigma-Aldrich, St. Louis, MO, United States), incubated at room temperature for 5 min, washed with DPBS, and centrifuged at 1,200 × g for 5 min. The supernatant was discarded, and the pellet was resuspended in DMEM containing 10% FBS and 1% PS. The cells were seeded in 100 mm cell culture dishes at a density of 3,000 cells/cm^2^ and incubated at 37°C in 5% CO_2_ environment. The medium was replaced every 2–3 days, and cells were subcultured upon reaching 70–80% confluence, exhibiting a fibroblast-like morphology.

To evaluate their differentiation capacity, cAT-MSCs were cultured in media specific for adipogenesis, osteogenesis, and chondrogenesis (StemPro Differentiation Kits; Gibco). Differentiation into adipocytes, osteocytes, and chondrocytes was confirmed by staining the cells with Oil Red O, 1% Alizarin Red, and Alcian Blue (Sigma-Aldrich), respectively. Surface marker expression was analyzed by flow cytometry using a FACSAria II system (BD Biosciences). Cells were stained with monoclonal antibodies against CD44-FITC, CD45-FITC, CD29-PE, CD73-PE, and CD90-PE (BD Biosciences). Fluorescence data were analyzed using FlowJo software (Tree Star, Woodburn, OR, United States).

### 2.3 Spheroid formation

cAT-MSCs were seeded at a density of 2 × 10^6^ cells per well in a Stem FIT 3D cell culture dish (c253000, MicroFIT, Inc., Ha-Nam, Republic of Korea). The cells were incubated in DMEM containing 20% FBS and 1% PS in a 5% CO_2_ environment at 37°C. Spheroid formation was monitored every 12 h for 48 h under an inverted microscope (Olympus CKX53; Olympus, Tokyo, Japan) at 4 × magnification. Spheroid areas were measured using ImageJ software (National Institutes of Health, United States, http://imagej.nih.gov/ij/).

### 2.4 Histological analysis of spheroid

After 48 h of culture, spheroids were collected by gentle pipetting, excluding any broken spheroids. The spheroids were washed three times with PBS and fixed with neutral-buffered 10% formalin overnight at 4°C. The fixed spheroids were embedded in Histogel (Epredia, Kalamazoo, MI, United States), followed by paraffin embedding, sectioning, and staining with hematoxylin (Thermo Fisher Scientific) and Eosin Y (Thermo Fisher Scientific).

### 2.5 Immunofluorescence analysis using whole spheroid staining

After fixation in 2.4 sections, the fixed spheroids were washed three times with 0.1% Triton X (Sigma-Aldrich) in DPBS (0.1% PBSTX) and permeabilized with 0.5% Triton X in DPBS (0.5% PBSTX) for 2 h at room temperature (RT). Spheroids were then washed three times with 0.1% PBSTX and blocked with 2% bovine serum albumin (Sigma-Aldrich) in 0.1% PBSTX for 1 h at RT. The spheroids were incubated overnight at 4°C with OCT4 rabbit pAb (1:100; ABclonal Technology, Boston, MA, United States), and SOX2 rabbit pAb (1:100; ABclonal Technology). After washing, the spheroids were incubated with goat anti-rabbit antibody conjugated with Alexa Fluor 488 (1:1000; Thermo Fisher Scientific) After incubation, the spheroids were washed three times with DPBS and mounted in an antifade mounting medium with 4′,6-diamidino-2-phenylindole (DAPI; Vector Laboratories, Burlingame, CA, United States). The spheroids were analyzed using a confocal laser scanning microscope (CLSM) (LSM 710; Zeiss, Oberkochen, Germany).

### 2.6 Western blot analysis

Protein extraction from 2D- and 3D-cultured cAT-MSCs was performed using the PRO-PREP Extraction Solution (iNtRON Biotechnology) according to the manufacturer’s instructions. Protein concentrations were determined using a DC Protein Assay Kit (Bio-Rad, Hercules, CA, United States). For protein expression analysis, 10 μg of protein was separated by 10% sodium dodecyl sulfate-polyacrylamide gel electrophoresis (SDS-PAGE) and transferred to polyvinylidene difluoride membranes (EMD Millipore, Burlington, MA, United States). Membranes were blocked with 5% skim milk in Tris-buffered saline and incubated overnight at 4°C with primary antibodies against SOX2 (1:1000; ABclonal Technology,), OCT4 (1:1000; ABclonal Technology), and *β*-actin (1:1000, Santa Cruz Biotechnology, Dallas, TX, United States). After washing, membranes were incubated with either goat anti-mouse or goat anti-rabbit horseradish peroxidase-labeled secondary antibodies (Bethyl Laboratories, Montgomery, TX, United States, and Enzo Life Sciences) at RT for 1 h. Immunoreactive bands were visualized by chemiluminescence (Advansta, San Jose, CA, United States) and imaged using an ImageQuant Las4000 mini (GE Healthcare Life Sciences, Chicago, IL, United States). Protein levels were normalized to *β*-actin.

### 2.7 Quantitative reverse transcription polymerase chain reaction measurement

Total RNA from 2D- and 3D-cultured cAT-MSCs was extracted using an Easy-Blue Total RNA Extraction Kit (iNtRON Biotechnology, Seongnam, Republic of Korea) according to the manufacturer’s instructions. RNA concentration and purity were measured using a spectrometer (Implen, Westlake Village, CA, United States). cDNA synthesis was performed using 1 μg of total RNA with the CellScript All-in-One 5 × 1st cDNA Strand Synthesis Master Mix (CellSafe, Yong-In, Republic of Korea). qRT-PCR was carried out in triplicate using 10 μL AMPIGENE qPCR Green Mix Hi-ROX with SYBR Green dye (Enzo Life Sciences, Farmingdale, NY, United States), 1 μL of cDNA, and 400 nM of forward and reverse primers (BIONICS, Seoul, Republic of Korea). Gene expression was normalized to glyceraldehyde 3-phosphate dehydrogenase (GAPDH). Primer sequences are listed in Table 1.

**Table 1:** Sequences of PCR primers used in this study.

Target gene	Primer	Sequence (5′ > 3′)	Reference
*GAPDH*	Forward	TTA ACT CTG GCA AAG TGG ATA TTG T	Yi et al. (42)
	Reverse	GAA TCA TAC TGG AAC ATG TAC ACC A	
*TGF-β1*	Forward	CTC AGT GCC CAC TGT TCC TG	Kim et al. (43)
	Reverse	TCC GTG GAG CTG AAG CAG TA	
*HIF-1α*	Forward	ATG ATG GTG ACA TGA TTT ACA TTT CT	Kim et al. (43)
	Reverse	GTA TTC TGC TCT TTA CCC TTT TTC AC	
*TSG-6*	Forward	TCC GTC TTA ATA GGA GTG AAA GAT G	Yi et al. (42)
	Reverse	AGA TTT AAA AAT TCG CTT TGG ATC T	
*COX2*	Forward	GCC TTA CCC AGT TTG TGG AA	Alder et al. (44)
	Reverse	AGC CTA AAG CGT TTG CGA TA	
*IL-1β*	Forward	AGT TGC AAG TCT CCC ACC AG	Manning et al. (45)
	Reverse	TAT CCG CAT CTG TTT TGC AG	
*IL-6*	Forward	ATG ATC CAC TTC AAA TAG TCT ACC	Yi et al. (42)
	Reverse	AGA TGT AGG TTA TTT TCT GCC AGT G	
*IL-10*	Forward	TGG CCC AGA AAT CAA GGA GC	Yi et al. (42)
	Reverse	CAG CAG ACT CAA TAC ACA CT	
*VEGF-A*	Forward	GAA TGC AGA CCA AAG AAA GAT AGA G	Kim et al. (43)
	Reverse	GAT CTT GTA CAA ACA AAT GCT TTC TC	
*bFGF*	Forward	ACT GGC TTC TAA ATG TGT TAC TGA C	Kim et al. (43)
	Reverse	TAG CAG ACA TTG GAA GAA AAA GTA T	

### 2.8 Enzyme-linked immunosorbent assay

The concentration of PGE2 in each cell culture supernatant was measured using a PGE2 Enzyme-Linked Immunosorbent Assay Kit (Enzo Life Sciences, Farmingdale, NY, United States) following the manufacturer’s instructions.

### 2.9 Macrophage polarization analysis

To compare the anti-inflammatory effects of conditioned media (CM) from 2D- and 3D-cultured cAT-MSCs, as well as dexamethasone, CM was collected from 2D cultures when the cells reached 80% confluence and from 3D cultures after 48 h. The media were centrifuged at 3000 rpm for 10 min at 4°C to remove cell debris and then stored at −70°C until use. DH82 cells were seeded in 6-well plates at a density of 1 × 10^6^ cells/well and treated with 200 ng/mL lipopolysaccharide (LPS) (Sigma-Aldrich, United States) for 24 h. Afterward, DH82 cells were treated with CM diluted 1:1 with DMEM containing 10% FBS and 1% PS, or with dexamethasone. After 48 h, macrophage polarization was assessed by flow cytometry using antibodies against CD86 (clone GL-1, APC conjugate; Tonbo Biosciences) and CD206 (clone C068C2, Alexa 488 conjugate; BioLegend). Cells were analyzed using a BD LSR flow cytometer, and data were processed using FlowJo software (Tree Star, Woodburn, OR, United States). Differences in the expression of TNFα, IL-1β, and IL-6 among the groups were evaluated by qRT-PCR.

### 2.10 Statical analysis

Each experiment was performed at least three times. GraphPad Prism (version 9.3.1) (GraphPad Software, San Diego, CA, United States; RRID:SCR_002798) was used for statistical analyses. One-way analysis of variance (ANOVA) was used to analyze the data, and Tukey’s multiple comparison test was performed. The findings are shown as mean plus standard deviation (SD). Differences were considered statistically significant at *p* < 0.05.

## 3 Results

### 3.1 Differentiation and characterization of cAT-MSCs isolated from canine adipose tissue

**Figure 1:**
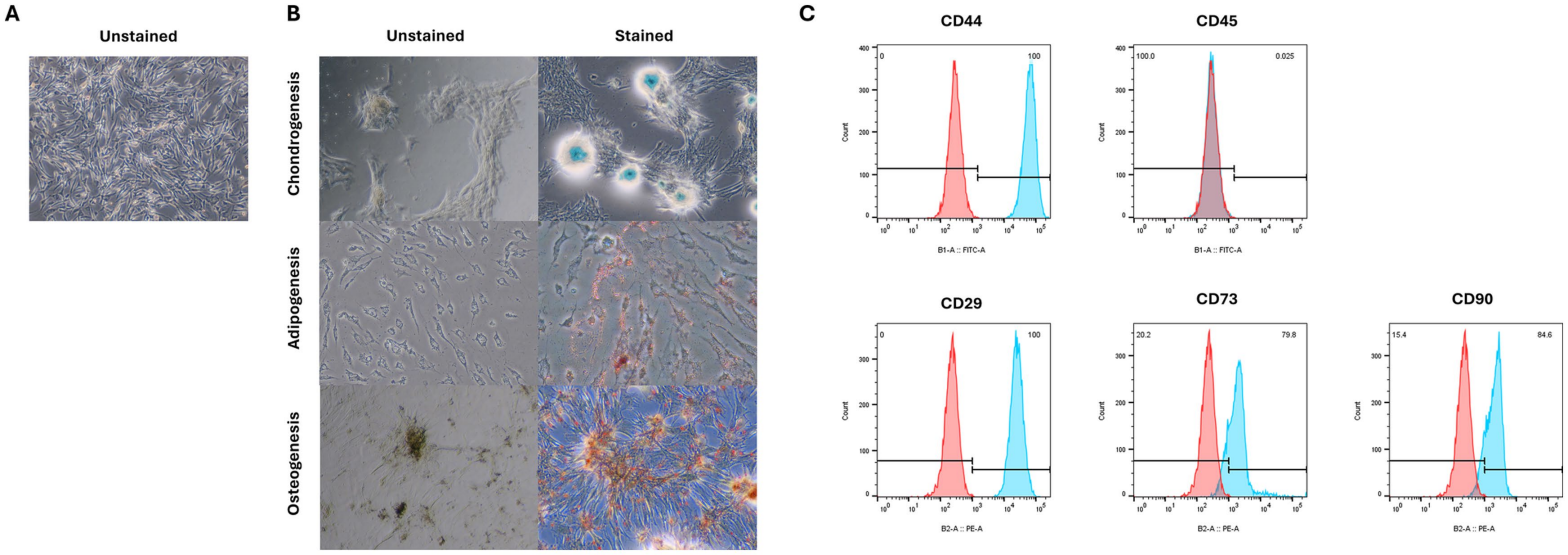
Characterization of cAT-MSC isolated from canine adipose tissue. **(A)** unstained cAT-MSC. **(B)** Differentiation into adipocyte, osteoblast, and chondrocyte was confirmed via Oil Red O, Alizarin Red, and Alcian Blue staining, respectively. **(C)** Flow cytometry analysis of cell surface markers. cAT-MSCs expressed CD29, CD44, CD 73 and CD90 and lacked CD45. (Blue: stained marker, red: unstained negative control).

We confirmed that the cells used in the experiment were cAT-MSCs by assessing their differentiation potential and surface marker expression. The cells successfully differentiated into adipocytes, osteoblasts, and chondrocytes, as indicated by positive staining with Oil Red O, Alcian Blue, and Alizarin Red S, respectively (Figure 1B). Additionally, flow cytometry analysis showed that the cells expressed the stem cell markers CD44, CD29, CD73, and CD90, while lacking expression of the hematopoietic marker CD45 (Figure 1C). These results confirm that the cells used in the experiments were indeed cAT-MSCs.

### 3.2 cAT-MSC spheroid formation and analysis

**Figure 2:**
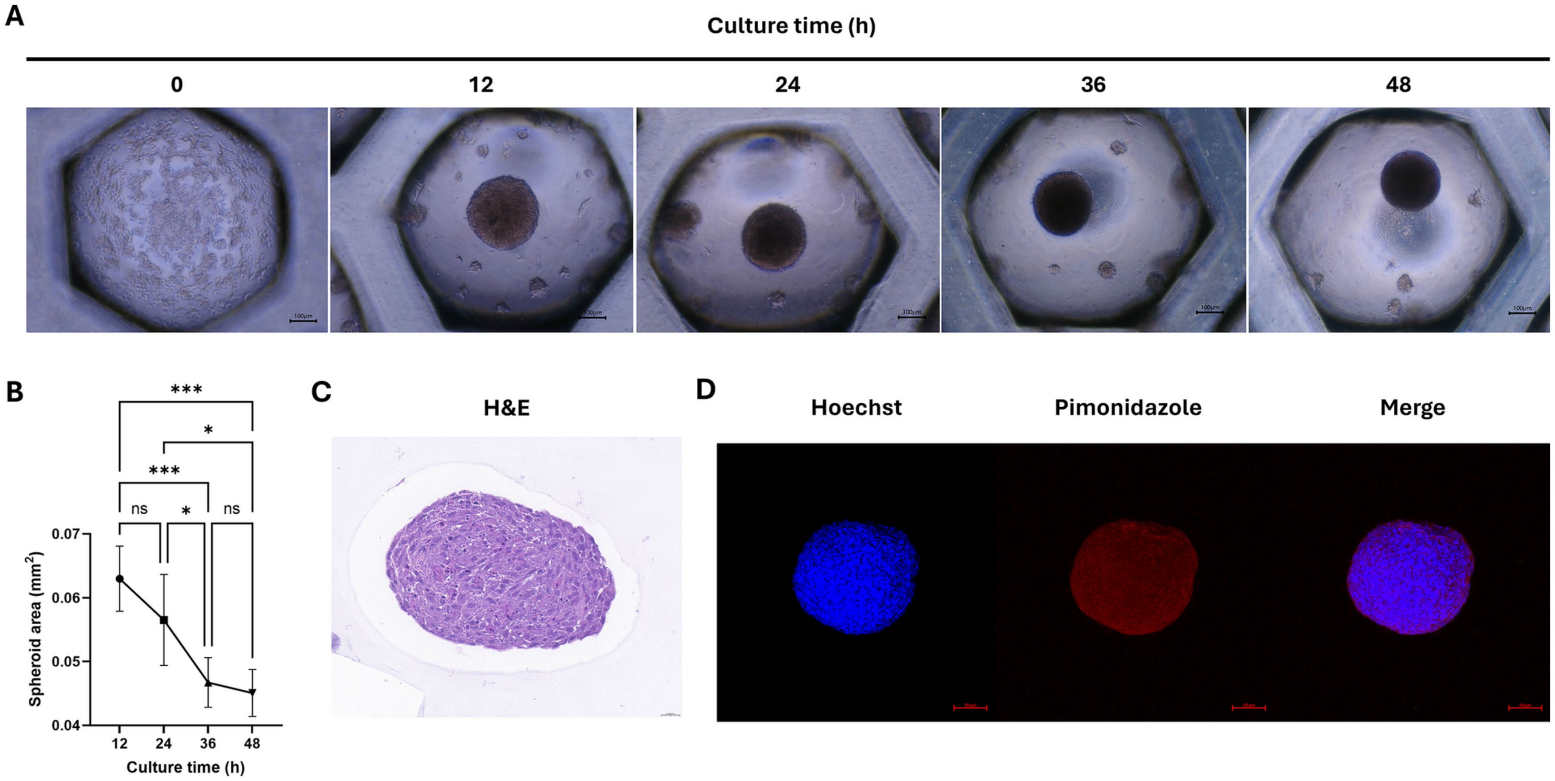
Analysis of cAT-MSCs spheroid formation. **(A)** Images of cAT-MSCs spheroids taken at 12-h intervals over 48 h. **(B)** Analysis of spheroid area over 48 h using ImageJ. The size of cAT-MSCs spheroids significantly decreased within 48 h. **(C)** cAT-MSCs spheroids were harvested at 48 h, sectioned, and stained with H&E staining (scale bar = 0.020 mm). **(D)** Immunohistochemistry analysis of cAT-MSCs spheroids harvested at 48 h. Spheroids were stained with APC dye-conjugated IgG1 mouse monoclonal anti-pimonidazole antibody (red) and Hoechst 33342 (blue) (scale bar = 100 μm). ^*^p < 0.05 and ^***^p < 0.001, as determined by one-way ANOVA.

We aimed to form spheroids using cAT-MSCs. For this purpose, we cultured 2 × 10^6^ stem cells in a Stem FIT 3D cell culture dish and imaged them at 12 h intervals (Figure 2A). The results showed that stem cells began to aggregate into spheroids as early as 12 h, with the size of the spheroids significantly decreasing after 36 h (Figure 2B). Spheroids were harvested at 48 h, sectioned, and stained with H&E. As shown in Figure 2C, the stem cells within the spheroids aggregated well. Additionally, to determine whether hypoxic conditions were induced during spheroid formation in the 3D culture, we used pimonidazole staining and confirmed the presence of hypoxic conditions (indicated by red staining) (Figure 2D).

### 3.3 Stemness-related protein expression in cAT-MSCs spheroids

**Figure 3:**
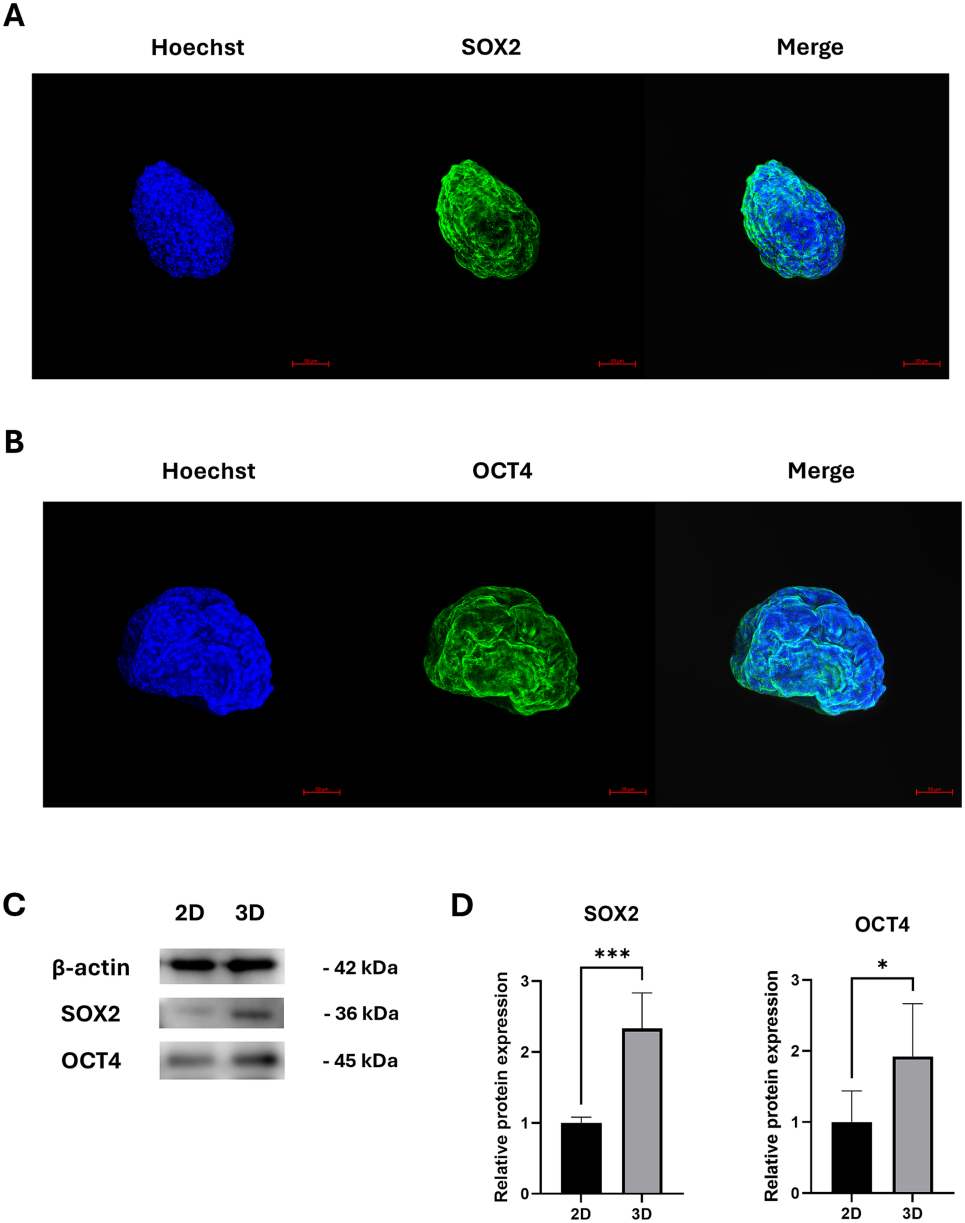
Stemness-related protein expression in cAT-MSCs spheroids. **(A)** cAT-MSCs spheroids harvested 48 h after incubation and stained with anti-SOX2 antibody (green) and Hoechst 33342 (blue) were examined using a confocal microscope (scale bar = 50 μm). **(B)** cAT-MSCs spheroids harvested 48 h after incubation and stained with anti-OCT4 antibody (green) and Hoechst 33342 (blue) were examined using a confocal microscope (scale bar = 50 μm). **(C)** SOX2 and OCT4, and TSG6 were extracted from 2D-cultured cAT-MSCs and 3D-cultured cAT-MSCs spheroids and quantified using western blotting. **(D)** Relative protein expressions in 2D and 3D-cultured cAT-MSCs were analyzed using ImageJ. SOX2 and OCT4 expression was significantly increased in 3D-cultured cAT-MSCs spheroids compared to 2D-cultured cAT-MSCs. ^*^p < 0.05 and ^***^p < 0.001, as determined by Student’s t-test.

Previous studies have reported that the expression of SOX2 and OCT4 in MSCs increases under hypoxic conditions (20, 21). To determine whether SOX2 and OCT4 expressions are similarly elevated under hypoxic conditions in cAT-MSC spheroids, we performed fluorescence staining and confirmed SOX2 and OCT4 expression using confocal imaging (Figures 3A,B). Furthermore, compared to 2D cultures, the expression of SOX2 and OCT4 was significantly increased in cAT-MSC spheroids, as verified by western blotting (Figures 3C,D).

### 3.4 Inflammation-related factor expression in 2D-cultured cAT-MSCs and 3D-cultured cAT-MSC spheroids

**Figure 4:**
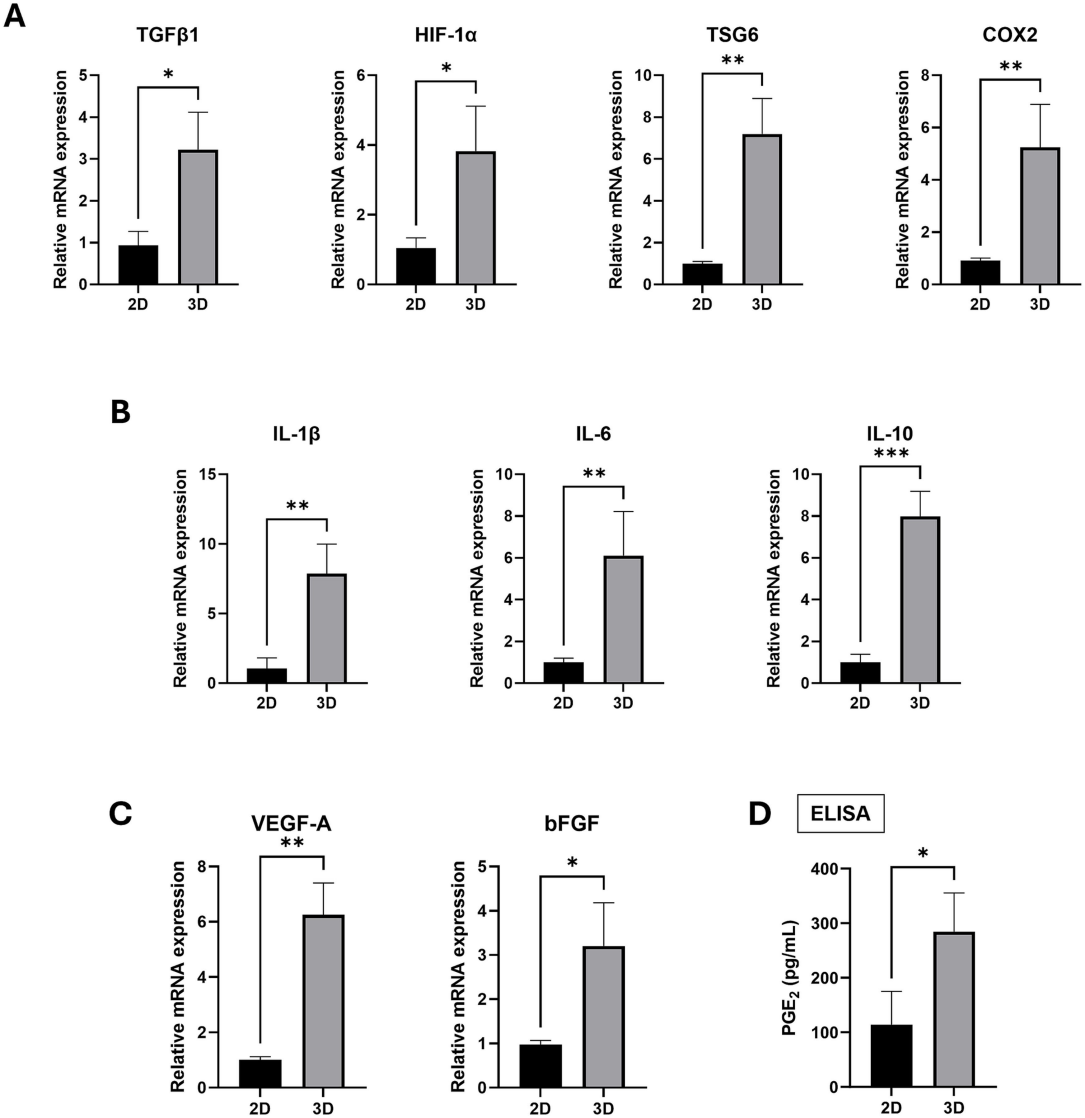
Inflammation and angiogenesis factor expression in 2D-cultured cAT-MSCs and 3D-Cultured cAT-MSCs Spheroids. The expression level of factors were measured by RT-PCR and ELISA. The expression of inflammation-related factors (**A**; TGFβ1, HIF1-*α*, TSG6, COX2), cytokines (**B**; IL-1β, IL-6, IL-10) and Angiogenesis (**C**; VEGF-A, bFGF) were significantly increased in 3D-cultured cAT-MSCs spheroids compared to 2D-cultured cAT-MSCs. (D) PGE2 ELISA results in the culture media of 2D-cultured cAT-MSCs and 3D-cultured cAT-MSCs spheroids. The PGE2 concentration was significantly higher in cAT-MSCs spheroids than in 2D-cultured cAT-MSCs. *p < 0.05 and **p < 0.01, as determined by Student’s t-test.

Given that the overexpression of SOX2 and OCT4 in MSCs has been associated with enhanced anti-inflammatory effects (22), we examined the gene expression of inflammation-related factors in spheroid-cultured cAT-MSCs. The mRNA levels of TGFβ1, HIF1α, TSG-6, and COX-2 were found to be elevated in 3D cultures compared to the 2D cultures (Figure 4A). Similarly, the mRNA levels of the inflammatory cytokines IL-1β, IL-6, and IL-10 were also increased in 3D cultures compared to the 2D cultures (Figure 4B). Additionally, the angiogenesis and tissue regeneration factors VEGF-A and bFGF exhibited higher mRNA levels in 3D cultures compared to the 2D cultures (Figure 4C). Finally, there was an increase in PGE2 protein expression in 3D cultures compared to the 2D cultures (Figure 4D).

### 3.5 Treatment of CM of 3D-cultured cAT-MSCs reduces LPS-induced inflammation in DH82

As shown in Figure 4, the gene expression of inflammation-related factors was higher in 3D-cultured cAT-MSC compared to 2D cultured cAT-MSC. We, therefore, investigated the immunomodulatory effects of CM from 3D-cultured cAT-MSCs on LPS-induced macrophages (DH82 cells). DH82 cells were initially treated with 200 ng/mL LPS for 24 h, followed by additional 48-h incubation with a 1:1 mixture of the original culture medium and CM. We then evaluated macrophage polarization and the expression of pro-inflammatory cytokines.

**Figure 5:**
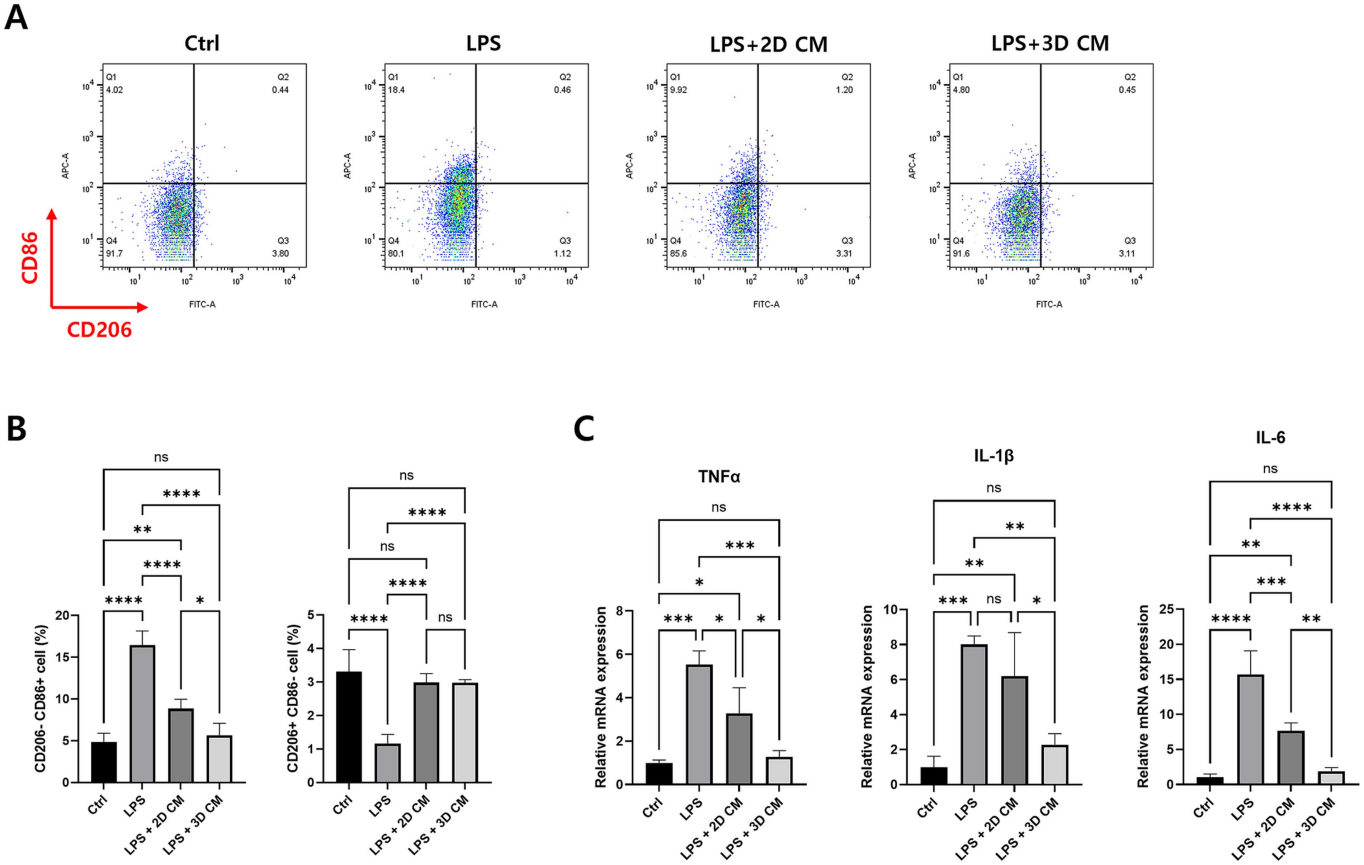
Treatment of conditioned media of 3D-cultured cAT-MSCs reduces LPS-induced inflammation in DH82 cells. DH82 cells were treated with 0.1 uM dexamethasone, conditioned media (CM) of 2D-or 3D-cultured cAT-MSCs for 48 h after LPS treatment. Inflammatory response changes were then determined by flow cytometry and RT-PCR. **(A)** Scatter plots of DH82 cells. M1 was identified as CD206-CD86+ cells, and M2 was identified as CD206 + CD86-cells. **(B)** Percentages of M1 (CD206-CD86+) to M2 (CD206 + CD86-) determined by flow cytometry. M1 polarization was significantly reduced in DH82 cells treated with 3D-cultured cAT-MSCs CM compared to those treated with LPS alone. **(C)** Differences in mRNA expression of inflammatory cytokines in DH82 cells. The expression of TNFα, IL-1β, and IL-6 was significantly decreased in 3D-cultured cAT-MSCs CM-treated DH82 cells compared to those treated with LPS alone. ^*^p < 0.05, ^**^p < 0.01, ^***^p < 0.001, ^****^p < 0.0001, as determined by one-way ANOVA.

The results showed that M1 polarization was significantly reduced in the group treated with CM from 3D-cultured cAT-MSC compared to the group treated with LPS alone. However, no significant changes were observed in M2 polarization. Interestingly, there were no significant changes in M1 polarization when comparing the group treated with CM from 3D-cultured cAT-MSCs to the group treated with CM from 2D-cultured cAT-MSCs (Figures 5A,B). Nevertheless, a significant decrease in the expression of pro-inflammatory cytokines TNFα, IL-1β, and IL-6 was observed in the group treated with CM from 3D-cultured cAT-MSCs compared to both the LPS alone-treated group, and the group treated with CM from 2D-cultured cAT-MSC (Figure 5C).

## 4 Discussion

SOX2 and OCT4 are essential transcription factors crucial for maintaining stem cell pluripotency and self-renewal. These markers are deeply involved in key developmental pathways and cellular differentiation processes, underscoring their significance in stem cell biology (23). Previous studies have shown that overexpression of SOX2 and OCT4 significantly enhances the viability, stemness, adipogenesis, osteogenesis, and proliferation of adipose-derived MSCs *in vitro* (24). Moreover, the adipogenic differentiation potential of human adipose-derived stem cells is improved in 3D cultures compared to 2D cultures (25). The ability of 3D cultures to mimic the *in vivo* microenvironment is the primary factor driving the enhanced expression of pluripotency genes and differentiation potential, with SOX2, OCT4, and NANOG being key stemness-related markers (26, 27). Recent studies demonstrated that Wharton’s jelly MSCs cultured in 3D microwell- and ULA-based systems exhibited significantly higher levels of OCT4 and SOX2 than those cultured in 2D (28). Similarly, our study found that the expression of the stemness markers SOX2 and OCT4 was significantly higher in 3D-cultured cAT-MSCs compared to 2D-cultured cAT-MSCs.

The immunomodulatory capacity of MSCs is central to their therapeutic potential, particularly in treating inflammatory and autoimmune diseases. Transitioning to 3D culture systems has been associated with a significant upregulation of immunomodulatory genes and proteins. This enhancement likely stems from the improved cell–cell and cell-ECM interactions in the 3D culture environment, which more accurately replicate *in vivo* conditions (29) and are crucial for maintaining stem cell properties and modulating the expression of factors that contribute to their therapeutic potential (30). Previous reports have indicated that the aggregation of MSCs in 3D cultures significantly enhances the expression of inflammation-related factors such as HIF-1α, TGFβ1, TSG-6, COX2, IL-1β, IL-6, IL-10 and PGE2 (31–35). In particular, PGE2 secreted by MSC spheroids plays a role in the anti-inflammatory effect by switching the phenotype of LPS-activated macrophages from M1 to M2. This process involves upregulation of IL-1 signaling, including IL-1α, IL-1β, and IL-1R1, in MSC spheroids, which promotes the secretion of COX2, PGE2, and TSG6 (14, 36). Furthermore, previous studies have shown that PGE2 secreted by MSCs exhibits anti-inflammatory effects in inflammatory disease models such as acute liver injury and colitis (37–39). Therefore, the increased expression of PGE2 in 3D-cultured cAT MSCs in this study suggests that they may have superior immunomodulatory effects compared to 2D-cultured cAT MSCs.

In our study, quantitative analysis revealed a significant increase in mRNA and protein expression levels of these factors in ULA-based 3D-cultured cells compared to 2D-cultured cells, likely due to internal hypoxic conditions. This upregulation underscores the role of the 3D microenvironment in enhancing the immunomodulatory profile of MSCs (8).

The therapeutic potential of MSCs is largely attributed to their paracrine activity, where factors secreted in the CM influence the surrounding cellular environment. Recent studies have shown that CM from 3D-cultured MSCs exhibits enhanced immunomodulatory effects compared to CM from 2D cultures. This enhancement is reflected in a higher concentration of extracellular vesicles (EVs) with increased complexity, as well as the secretion of anti-inflammatory cytokines, such as IL-10, IL-4, TGFβ, and LIF, and growth factors, such as VEGF and FGF, which are involved in tissue regeneration and immune modulation (8, 40, 41). The immunomodulatory effects of 3D CM are believed to results from the physiologically relevant environment provided by 3D culture, leading to a secretion profile enriched in cytokines and growth factors that reduce the polarization of macrophages toward an inflammatory phenotype (14, 31). For instance, MSC-CM has shown therapeutic potential in conditions such as diabetic polyneuropathy, demonstrating improvements through anti-inflammatory, neuroprotective, and angiogenic effects (29). The clinical application of MSCs and their secretomes, including EVs isolated from MSC-CM, holds promise for treating diseases characterized by inflammation, such as acute respiratory distress syndrome and pneumonia. The comparative effectiveness of 3D CM with corticosteroids in reducing inflammation underscores its potential as a safer and more natural alternative for modulating immune responses in various diseases. The potential of MSC-CM as a cell-free therapy for inflammatory conditions, offering advantages over traditional anti-inflammatory drugs, presents a promising avenue for future studies.

## 5 Conclusion

This study demonstrates that 3D-cultured cAT-MSCs retain their stemness and exhibit enhanced immunomodulatory effects, as evidenced by the significant upregulation of immunomodulatory factors and the reduction in the pro-inflammatory macrophage phenotype. We suggest that 3D-cultured cAT-MSCs hold promise as a novel treatment for inflammatory diseases and could offer an alternative to traditional anti-inflammatory drugs. These findings underscore the need for further research to validate the therapeutic potential of 3D MSC culture systems, explore their efficacy in various inflammatory conditions, and investigate their translation into clinical practice.

## Data Availability

The original contributions presented in the study are included in the article/supplementary material, further inquiries can be directed to the corresponding author.
